# Systematic review of the surgical treatment for symptomatic os acromiale

**DOI:** 10.4103/0973-6042.80461

**Published:** 2011

**Authors:** Joshua D. Harris, Michael J. Griesser, Grant L. Jones

**Affiliations:** Department of Orthopaedics, The Ohio State University Sports Medicine Center, Columbus, Ohio, USA

**Keywords:** Acromionectomy, acromioplasty, excision, internal fixation, os acromiale

## Abstract

The optimal surgical treatment for symptomatic os acromiale that has failed nonoperative management is unclear in the literature. We conducted a systematic review of multiple medical databases for level I–IV evidence. Both radiographic and clinical outcomes were analyzed. Nine studies met the inclusion criteria (118 subjects, 125 shoulders). One hundred and fifteen subjects were treated surgically (122 shoulders). The mean age of the subjects was 49±11 years. The mean preoperative duration of symptoms was 12±8.6 months. Mesoacromiale was the most common type treated (94%). Internal fixation was the most common surgical technique used (60%), followed by excision (27%) and acromioplasty (13%). Rotator cuff repair was the most common concurrent surgical technique (performed in 59% of the surgically treated shoulders), followed by distal clavicle excision (25%). All surgical techniques resulted in improvement in clinical outcomes. Surgical management of symptomatic os acromiale that has failed nonoperative measures may predictably lead to improved outcomes.

## INTRODUCTION

**Figure 1:**
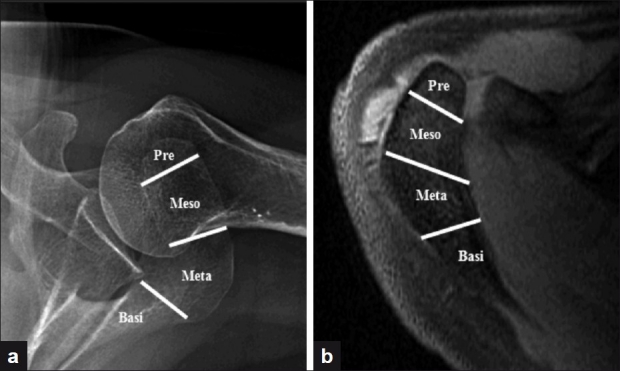
(a) Axillary radiograph; (b) Axial magnetic resonance image. (Pre – Preacromiale; Meso – Mesoacromiale; Meta – Metaacromiale; Basi – Basiacromiale)

Appearance of the acromial centers of ossification (preacromion, mesoacromion, metaacromion and basiacromion) occurs between 15 and 18 years of age and should complete by 25 years of age.[1] An os acromiale is a failure of fusion at one of the junctions of these ossification centers. The incidence of the os acromiale ranges from 1% to 30%.[2] Bilateral involvement may occur in 33%[3–4]–62%[5] of the cases. The most common nomenclature denotes naming of the os acromiale by the fragment anterior to the unfused segments (hence, an ununited mesoacromion and metaacromion is called a mesoacromiale) [Figure 1]. Thus, the bone anterior to the site of the two most anterior unfused segments is called “preacromiale.” The mesoacromiale is the most common os acromiale type, followed by preacromiale and then metaacromiale.[3–4]

Clinical manifestations of symptomatic os acromiale involve impingement-like pain, night pain and tenderness at the site of the ununited fragments. Pathomechanisms involved include excessive motion at this ununited site, predisposing to subacromial impingement and, potentially indirectly, rotator cuff tears and also degenerative pseudarthrosis changes with cyst formation. The “false-articulation” may actually be like a true synovial joint with articular cartilage[6] or may be a fibrous synchondrosis.[7] Diagnosis of symptomatic os acromiale may preclude allowance of time in patients less than 25 years of age. Nevertheless, this diagnosis has been made in age ranges from teenagers[8] to the elderly.[9] Nonsurgical management has traditionally been the initial treatment of choice in all cases of os acromiale.[2] Failure of nonoperative measures may require surgical intervention. Surgical options include excision of the os acromiale fragment, subacromial acromioplasty and internal fixation. The high incidence of concurrent rotator cuff tear warrants consideration during rotator cuff repair.[2] Although concurrent symptomatic acromioclavicular (AC) joint osteoarthritis has been reported,[10] the AC joint is a stabilizing structure for a mesoacromiale; thus, its removal during a distal clavicle excision would further destabilize the os acromiale. In addition, given that the origin of vascularity for the anterior acromion is anteromedial,[2] a distal clavicle excision may compromise the requisite blood supply to the anterior os acromiale fragment, should fixation be attempted, increasing the risk of nonunion. Small pre os acromiale fragments generally do well with excision and meticulous deltoid repair. Large unstable meso- or meta-os acromiale fragments generally do poorly with excision/acromionectomy. The choice of fixation versus acromioplasty versus excision has yet to be demonstrated clearly in the literature for symptomatic, stable or unstable, pre-, meso- and meta-os acromiale.

The purpose of this review was to report and compare the clinical and radiographic outcomes following surgical management of symptomatic os acromiale. We hypothesized that (1) excision of pre os acromiale leads to improved clinical outcomes, (2) arthroscopic excision of meso- and meta-os acromiale fragments leads to improved clinical outcomes while open excision (without deltoid repair) leads to poor clinical outcomes, (3) arthroscopic acromioplasty of stable os acromiale leads to improved clinical outcomes and (4) stable fixation of meso- and meta-os acromiale fragments leads to improved clinical outcomes.

### Methods

To address our hypotheses, we performed a systematic review of the available medical literature using several medical databases, including Pubmed, MEDLINE, CINAHL (Cumulative Index to Nursing and Allied Health Literature), SPORTDiscus with full text and Cochrane Central Register of Controlled Trials/Database of Systematic Reviews/Methodology Register. The search was independently performed by all three authors (JDH, MJG, GLJ) on August 7, 2010. Database journal search dates ranged from 1950 to the current day. Search terms included “os acromiale,” “fixation,” “excision” and “repair.” Levels I, II, III and IV evidence (according to the Oxford Centre for Evidence-Based Medicine used by the American version of the Journal of Bone and Joint Surgery) were applied.[11] Potential inclusive papers and their bibliographies were manually reviewed and discussed among authors and a decision was made regarding the inclusion or exclusion. In the event of disagreement among authors for study inclusion, the final decision was made by the senior author (GLJ). The full text article was reviewed and the reference list was checked for potential studies not identified by our original search.

### Inclusion criteria

The inclusion criteria were:

- Level I, II, III and IV evidence studies
- English language studies
- Human subjects
- Study publication date from January 1, 1950 to August 7, 2010
- Studies investigating clinical outcomes following treatment of symptomatic os acromiale
- Studies investigating nonoperative management of symptomatic os acromiale
- Studies investigating operative management of symptomatic os acromiale, including fragment excision/acromioplasty/acromionectomy and internal fixation/repair
- Studies with a minimum mean follow-up of 24 months

### Exclusion criteria

The exclusion criteria were:

- Level V evidence studies and Level IV evidence isolated patient/subject case reports
- Non-English language
- Basic science, animal model, biomechanical studies
- Studies not reporting clinical outcomes following treatment of symptomatic os acromiale
- Studies investigating fixation or repair of iatrogenically separated (for visualization of subacromial space and rotator cuff repair) os acromiale
- Studies reporting only imaging (X-ray, ultrasound, computed tomography or magnetic resonance imaging) outcomes
- Studies with a mean follow-up of less than 24 months

Table 1 displays the search strategy citation results of all databases searched. Forty-two studies were initially retained and analyzed further for potential inclusion. One study was excluded due to non-English language (Turkish).[12] Twelve Level IV isolated subject case reports were excluded.[13–24]

**Table 1:** Database search citation strategy

	Pubmed	MEDLINE	CINAHL	SportDiscus	Cochrane
“os”+“acromiale”	62	50	0	17	0
“os”+“acromiale”+“excision”	8	0	0	0	0
“os”+“acromiale”+“fixation”	12	1	0	2	0
“os”+“acromiale”+“repair”	5	1	0	3	0

CINAHL – Cumulative Index to Nursing and Allied Health Literature; Cochrane – Cochrane Central Register of Controlled Trials; All databases were searched on August 7, 2010

Five imaging studies without clinical outcomes were excluded.[3,25–28] Two studies reported long-term outcomes following acromionectomy for multiple reasons (however, none for os acromiale) and were excluded.[29–30] Two studies reported an incidence of os acromiale (based on imaging and arthroscopy) with concurrent rotator cuff tear and did not report surgical treatment or clinical outcomes and therefore were excluded.[31–32] Three Level V evidence review studies were excluded.[2,4,33] Clinical follow-up of less than 24 months was found in two studies,[34–35] and they were excluded. Five studies did not report the length of follow-up and were excluded.[36–40] One study did not report any demographic data of surgically treated patients and therefore was excluded.[41] Nine studies met all inclusion criteria and were analyzed further.

Subject inclusion criteria varied across studies, although, typically, were based on subacromial impingement symptoms (with or without the use of diagnostic subacromial lidocaine injection) and tenderness to palpation at a mobile/unstable os acromiale. Nomenclature of os acromiale types were based on the anterior fragment (e.g., an os acromiale between the pre- and mes-acromion was defined as a pre os acromiale; an os acromiale between the mesoacromion and metaacromion was defined as a meso-os acromiale; an os acromiale between the metaacromion and basiacromion was defined as a meta-os acromiale). Surgical techniques were described in detail within each study. For purposes of this review, os acromiale excision was defined as total/complete removal of the os acromiale, either arthroscopic or open. Acromioplasty was defined as incomplete removal of the os acromiale (either via open Neer acromioplasty or arthroscopic subacromial cutting-block acromioplasty). Fixation was defined as open or percutaneous internal placement of hardware fixing the os acromiale fragment to the more proximal scapula/acromion. Clinical assessment tools used postoperatively included the UCLA (University of California at Los Angeles) shoulder evaluation form,[42–44] PENN shoulder score,[45–46] Constant score[47–48] and ASES score (American Shoulder and Elbow Surgeons).[49] All but one study used postoperative X-rays. Two studies quantitatively reported isokinetic strength testing,[8,48] while two more qualitatively reported postoperative strength.[42,44]

A Z-test for two proportions (one-tailed, with assumption of acceptance of null hypothesis equivalent) was used to compare the proportions from two independent groups to test for significant differences between the acromioplasty and fixation groups (patient satisfaction), screw and K-wire fixation groups (rate of radiographic union) and requirement for removal of hardware following screw and K-wire fixation groups. Statistical analysis was performed using a free online statistical calculator.[50]

## RESULTS

Nine studies met the inclusion criteria (all Level IV evidence).[8–9,42,44,47–49,10,51] Two studies denied the presence of a financial conflict of interest (COI),[9,48] while seven did not report the presence or absence of a COI.[8,42,44,47,49,10–51] One-hundred eighteen subjects were included (125 shoulders, seven bilateral cases). One-hundred fifteen subjects underwent surgical management of os acromiale (122 shoulders). Three subjects were managed nonoperatively (three shoulders). There were 82 males and 36 females. When reported (*n*=46), the right shoulder was involved 65% of the time (30/46) and, when reported (*n*=58), the dominant shoulder was involved 66% (38/58) of the time. The mean age of the subjects was 49±11 years (range, 18–73 years of age). The mean duration of the symptoms was 12±8.6 months (range, 2–36 months).

Meso-os acromiale was the most common type of os treated across all subjects in this review (94%). Internal fixation was the most common surgical technique (60%; 73/122), followed by excision (27%; 33/122) and acromioplasty (13%; 16/122). A “tension-band” technique with two parallel Kirschner wires and either stainless steel wire or suture in a cerclage or figure-of-eight technique was the most common fixation method (67%). Bone graft was used in 22 cases of fixation (30% of all fixation cases; 22/73; 16 iliac crest bone graft, six os graft). Rotator cuff repair was the most common concurrent surgical technique (59% of surgically treated shoulders; 72/122), followed by distal clavicle excision (25%; 31/122) and long-head biceps tenodesis (11%; 14/122). Table 2 displays the distribution of the surgical techniques, Table 3 displays the type of surgical fixation used and Table 4 displays the concurrent surgical techniques used in addition to the surgical management of the os acromiale.

**Table 2:** Surgical treatment distribution

OS acromiale type	Excision		Acromioplasty		Fixation	Total
	Open	Arthroscopic	Open	Arthroscopic	Open	
Pre	4	0	0	0	0	4
Meso	5	24	11	5	70	115
Meta	0	0	0	0	3	3

Subtotal	9	24	11	5	73	122

Total	33		16		73	

**Table 3:** Types of surgical fixation and postoperative radiographic healing

Type of surgical fixation	Number of subjects	Follow-up X-ray healing rate
“Tension-band” with K-wires	49	31/49 (63)
“Tension-band” with cannulated screws	20	19/20 (95)
Cannulated screws	4	4/4 (100)

K-wires – Two kirschner wires, diameter range 0.062 inch/1.57–2.5 mm; Cannulated screws – Includes two 3.5 mm and two 4.5 mm cannulated, partially threaded, Cancellous screws – Two 4 mm cannulated, partially threaded; Cortical screws – Two 4.5 mm, cannulated Herbert screws; Figures in parenthesis are in percentage

**Table 4:** Concurrent surgical techniques

Type of surgical technique	Number of subjects
Rotator cuff repair	72
Distal clavicle excision	31
Long-head biceps tenodesis	14
Rotator cuff debridement	8
Humeral head bone graft	6
SLAP repair	2
Latissimus dorsi transfer	1
Long-head biceps tenotomy	0

SLAP – Superior labrum anterior-to-posterior

All surgical techniques resulted in improvement in clinical outcomes. Table 5 displays the subject outcomes based on standardized clinical outcome measures. Table 6 displays the range-of-motion outcomes. Table 7 displays the individual study demographics.

**Table 5:** Clinical outcomes

Clinical outcome measure used	Surgical technique used (number of subjects)	Mean preoperative score	Final postoperative score
UCLA	Excision (13)	16	31 (29–34 months)
	Fixation (10)		
Constant	Excision (6)	nr	71 (41–44 months)
	Acromioplasty (5)		
	Fixation (37)		
PENN	nr	nr	nr
ASES	Fixation (6)	39	93 (55 months)

UCLA – University of California at Los Angeles; Constant – Constant Murley; PENN – PENN shoulder score; ASES - American Shoulder and Elbow Surgeons; nr – Not reported

**Table 6:** Range-of-motion outcomes

Surgical technique	Preoperative forward elevation	Postoperative forward elevation	Preoperative external rotation	Postoperative external rotation
Excision	nr	nr	nr	nr
Acromioplasty	111[10]	142 (40 months)	32	40 (40 months)
Fixation	116[10]	141 (40 months)	38	37 (40 months)
	117[51]	160 (29 months)	36	45 (29 months)
	117[49]	165 (55 months)	60	62 (55 months)

nr – Not reported; All values reported in degrees (°)

**Table 7:** Individual study demographics

Study	Number of subjects surgery	Number of shoulders surgery	M/F	R/L	Dom/nondom	Mean age (y) (range)	Pre	Meso	Meta	Mean follow-up (mo)	Confounding surgical techniques	Excision	Acromioplasty	K-wire fixation	Screw fixation
Pagnani *et al*. 2006[8]	9	11	9/0	nr	7/4	nr (18–25)	0	11	0	11	None	11	0	0	0
Abboud *et al*. 2006[10]	19	19	12/7	12/7	13/6	53 (35–73)	0	19	0	40	8 RCR	0	11	5	3
Ozbaydar *et al*. 2006[51]	6	6	1/5	4/2	nr	58.5 (51–64)	0	6	0	29	6 RCR, 1 LHBT tenodesis, 6 HHBG	0	0	2	4
Boehm *et al*. 2003[48]	33	33	23/10	nr	nr	56 (44–70)	3	30	0	41	33 RCR, 19 DCE, 9 LHBT tenodesis, 1 LT	6	5	22	0
Wright *et al*. 2000[42]	12	13	8/4	nr	nr	36 (18–54)	0	13	0	29	1 RCR, 4 RCD, 2 SLAP repair	13	0	0	0
Ryu *et al*. 1999[44]	4	4	3/1	nr	2/2	27 (20–43)	0	4	0	34	2 RCD	0	0	0	4
Satterlee *et al*. 1999[49]	6	6	4/2	4/2	3/3	48 (29–63)	0	6	0	55	3 RCR	0	0	0	6
Hertel *et al*. 1998[47]	12	15	12/0	10/5	11/4	54 (37–63)	0	15	0	44	15 RCR, 12 DCE, 4 LHBT tenodesis	0	0	15	0
Warner *et al*. 1998[9]	14	15	7/7	nr	nr	57 (19–76)	1	11	3	34	8 RCR	3	0	5	7

M – Male; F – Female; R – Right; L – Left; Dom - Dominant shoulder; Non-dom – Nondominant shoulder; y – Years; mo – Months; nr – Not reported; RCR – Rotator cuff repair; RCD – Rotator cuff debridement; LHBT – Long-head biceps brachii tendon; DCE – Distal clavicle excision; SLAP – Superior labrum anterior-to-posterior; LT – Latissimus dorsi muscle-tendon transfer

### Excision

#### Arthroscopic

No studies compared open and arthroscopic excision. Pagnani *et al*. performed the arthroscopic excision in 11 meso-os acromiales.[8] These patients, aged 18–25 years of age, were able to return to sport by 14 weeks postoperatively and demonstrated full isokinetic strength in abduction, internal rotation and external rotation versus the contralateral side. Wright *et al*. performed arthroscopic excision on 13 meso-os acromiales.[42] Although this cohort of patients was slightly older (mean, 36 years; range, 18–54 years), they all still demonstrated full anterior deltoid and rotator cuff strength by 6 months postoperatively, with 85% (11/13) patient satisfaction.

#### Open

Boehm *et al*.[48] and Warner *et al*.[9] performed open excision with deltoid re-attachment in nine os acromiales (four pre and five meso). In the former, patients (mean age, 56 years; range, 44–70 years) undergoing open os excision had equivalent Constant scores and patient satisfaction versus age- and gender-matched controls between those patients undergoing open Neer acromioplasty and fixation with either K-wire or cannulated screw tension-band constructs. Warner *et al*. (mean age, 57 years; range, 19–76 years) demonstrated a good outcome in a pre os excision and poor outcomes in both meso-os excisions (however, these were both following a failed internal fixation attempt).

### Acromioplasty

Abboud *et al*. performed arthroscopic cutting-block (*n*=5) and open Neer (*n*=6) acromioplasty in 11 patients with a stable meso-os.[10] This group of patients was compared retrospectively with a group of eight patients undergoing open reduction and internal fixation with either K-wires (*n*=5) or cannulated screws (*n*=3). Although patient satisfaction (defined by improved pain, motion and strength) was greater in the acromioplasty group (7/11; 64%) versus the fixation group (3/8; 38%), the difference was not significant (Z=0.655; 95% confidence level). Boehm *et al*. also retrospectively compared patients undergoing open Neer acromioplasty versus internal fixation with K-wires.[48] Age- and gender-matched control comparison demonstrated equivalent Constant scores and patient satisfaction among the groups.

### Fixation

Surgical fixation with cannulated screws led to a significantly greater rate of radiographic healing (96%; 23/24) versus fixation with Kirschner wires (63%; 31/49) (Z=2.735; 99.7% confidence level). The rate of radiographic healing correlated with significantly improved clinical outcome (UCLA score, ASES scores and patient satisfaction). Following screw fixation, Ryu *et al*.[44] demonstrated full strength and motion with normal UCLA score (35/35) in four of four patients (all radiographically united by 10–16 weeks postoperatively). Following screw fixation, Satterlee *et al*.[49] also demonstrated excellent ASES scores in six of six patients (all with radiographic union). Warner *et al*.[9] showed a significantly better union rate (86% versus 20%; 6/7 versus 1/5; Z=1.682; 95% confidence level) (mean 9-week time to union; range 7–20 weeks) following screw versus K-wire fixation. In this study, the union group demonstrated good clinical outcomes in six of the seven cases (86%), with the one patient experiencing a poor clinical outcome because of repeat rotator cuff tear rather than failure of fixation, while in the nonunion group, all patients demonstrated poor clinical outcome (five of five). Hertel *et al*.[47] demonstrated significantly greater Constant scores in patients with radiographic union versus nonunion (*P*=0.169). Ozbaydar *et al*.[51] demonstrated a significantly greater healing rate following screw versus K-wire fixation (four of four versus zero of two), with an overall improvement in the UCLA score from 11 to 28 (out of 35). Following K-wire fixation, Boehm *et al*.[48] showed a 68% union rate (15/22), although there was no difference between the union and nonunion groups with regard to Constant scores. Following both screw (*n*=3) and K-wire (*n*=5) fixation, Abboud *et al*.[10] showed a 100% union rate. Despite the latter, only three of the eight patients (38%; one screw and two K-wire) had a satisfactory clinical outcome (however, two of the five unsatisfied patients still had a good subjective rating). Requirement for removal of internal fixation hardware was significantly greater after Kirschner wire fixation cases (88%; 43/49) versus cannulated screw fixation cases (38%; 9/24) (Z=4.181; 100% confidence level).

### Surgical complications

There were four cases of deep infection and two cases of superficial infection following surgical treatment of os acromiale.[48] One case of deep infection followed open excision, two cases followed open acromioplasty and one case followed open reduction and internal fixation. All these four cases required surgical incision and drainage. Two cases of superficial infection followed open reduction and internal fixation. These two cases responded to nonoperative, antibiotic management. There were no other complications following surgical intervention.

## DISCUSSION

The optimal surgical treatment for symptomatic os acromiale that had failed nonoperative management was unclear in the literature. The purpose of this review was to report and compare the clinical and radiographic outcomes following surgical management of symptomatic os acromiale. We hypothesized that (1) excision of pre os acromiale leads to improved clinical outcomes, while excision of meso- and meta-os acromiale fragments leads to poor clinical outcomes, (2) arthroscopic acromioplasty of stable os acromiale leads to improved clinical outcomes and (3) stable fixation of meso- and meta-os acromiale fragments leads to improved clinical outcomes.

This systematic review has shown that both arthroscopic and open excision of preacromiale and mesoacromiale lead to improved clinical outcomes, with full strength and motion and reduced pain. Both arthroscopic and open acromioplasty of mesoacromiale lead to improved patient satisfaction, with reduced pain and greater strength and motion. There is no significant difference in clinical outcome between acromioplasty and internal fixation. Fixation of mesoacromiale with cannulated screws versus K-wires leads to a significantly greater rate of radiographic healing and improved clinical outcome. Further, fixation with cannulated screws versus K-wires leads to a significantly reduced need for removal of internal fixation hardware.

Os acromiale may become symptomatic secondary to mobility at the site of failed fusion of ossification centers in the acromion. On attempted shoulder motion, deltoid firing pulls on the mobile fragment and may lead to dynamic subacromial impingement. Three general surgical techniques (open and arthroscopic) have been described to treat an os acromiale in a symptomatic patient that has failed nonsurgical treatments: excision, acromioplasty and internal fixation.

Fragment excision (either open or arthroscopic) may be able to significantly improve clinical outcomes with a meticulous surgical technique. Prior studies have demonstrated unacceptably poor outcomes with large os fragment excision and not repairing the deltoid origin. Neer *et al*. reviewed 30 consecutive patients that had radical acromionectomy and no deltoid repair.[30] All the patients had poor clinical results, with persistent pain and marked weakness, and over 25% had a serious wound complication. Further, deltoid scarring predictably led to extreme difficulty in the revision of the surgical situation. Bosley reviewed 35 consecutive patients that had total acromionectomy and meticulous deltoid repair to the remaining acromion.[29] Pain, motion, strength, function and patient satisfaction were excellent in 71% and good in 11% of the patients. Further, in four of five patients with the least satisfactory results, there was a chronic, massive rotator cuff tear confounding. Our review has demonstrated that both arthroscopic excision (that avoids disruption of the deltoid origin) and open excision with meticulous deltoid repair may significantly improve the clinical outcomes.

Acromioplasty of a stable os acromiale (either open or arthroscopic) may significantly improve the clinical outcomes, equivalent to that of internal fixation and excision groups and to that of age- and gender-matched controls. This technique works by reducing the dynamic bony impingement of the rotator cuff and subacromial bursa on the undersurface of the acromion. Arthroscopic acromioplasty should avoid disruption of the deltoid origin and open acromioplasty, just like open os excision, should attempt to carefully repair the deltoid attachment. Further, acromioplasty of unstable, mobile fragments may not lead to enough reduction in the dynamic impingement process and lead to poor clinical results.

Fixation of os acromiale significantly improves the clinical outcomes. Clinical outcome and radiographic healing of the unfused fragments appear to be related, regardless of type of fixation used. Healed fragments have a better clinical outcome than those that go on to nonunion. Low subject numbers and the lack of subject-level-specific data within this review preclude proof of correlation. Fixation with cannulated screws led to greater radiographic healing rates than fixation with K-wires, as did clinical outcome, and the reduced need for removal of hardware. The biomechanical composition of screws versus K-wires accounts for this. A screw is a more rigid, stiffer construct with threads and a thread depth that reduces/eliminates hardware pullout versus a K-wire, which is either smooth or with threads with a very small thread depth, more easily allowing for pullout. This may lead to skin complications due to prominent hardware and persistent pain.

The findings within this systematic review are inherently limited by the weaknesses within each study, specifically Level IV evidence. These studies were all retrospective in nature, with only one study performing matched control analysis.[48] Sources of selection bias within this review include different numbers of subjects within each surgical group analyzed and small numbers of subjects within each surgical group analyzed. The presence of concurrent surgical interventions (e.g. rotator cuff repair [open or arthroscopic], distal clavicle excision [open or arthroscopic], long head biceps tendon tenodesis, the use of autologous bone graft) is a source of performance bias, as are the minor technique variations within each surgical group analyzed.

Nearly all of the patients within this review had failed nonsurgical management measures prior to surgical intervention. Nevertheless, the lack of a nonoperative control group to compare the surgical techniques precludes the true outcomes of the surgical procedure itself. Assessment of clinical outcomes using validated outcome measures with measurement by independent observers is necessary to minimize detection bias. In this review, these parameters were either not performed or not reported in each of the studies. Further, heterogeneity of the assessment tools used in this review prevent accurate comparison, as do the definitions of patient satisfaction, excellent, good, fair, etc. The optimal assessment of an orthopedic disease treatment is by using a body-part-specific outcome tool and a general health outcome tool. This review lacked any general health outcome tools and only reported three shoulder-specific tools (UCLA, ASES and Constant) outcomes. Further, one study reported the use of the PENN outcome tool, but failed to actually report the subjects’ PENN scores.[10]

## CONCLUSIONS

Surgical management of symptomatic os acromiale that has failed nonoperative measures may predictably lead to improved outcomes. Both arthroscopic and open excision of preacromiale and mesoacromiale lead to improved clinical outcomes, with full strength and motion and reduced pain. Both arthroscopic and open acromioplasty of mesoacromiale lead to improved patient satisfaction, with reduced pain and greater strength and motion. There is no significant difference in clinical outcome between acromioplasty and internal fixation. Fixation of mesoacromiale with cannulated screws versus K-wires leads to a significantly greater rate of radiographic healing and improved clinical outcome. Further, fixation with cannulated screws versus K-wires leads to a significantly reduced need for removal of internal fixation hardware.
